# Advances in the Extraction, Purification, Structural Characteristics and Biological Activities of *Eleutherococcus senticosus* Polysaccharides: A Promising Medicinal and Edible Resource With Development Value

**DOI:** 10.3389/fphar.2021.753007

**Published:** 2021-11-01

**Authors:** Xiaojie Li, Cai Chen, Aijing Leng, Jialin Qu

**Affiliations:** ^1^ Laboratory of Integrative Medicine, The First Affiliated Hospital of Dalian Medical University, Dalian, China; ^2^ Institute (College) of Pharmacy, Dalian Medical University, Dalian, China; ^3^ Institute (College) of Integrative Medicine, Dalian Medical University, Dalian, China; ^4^ Department of Traditional Chinese Medicine, The First Affiliated Hospital of Dalian Medical University, Dalian, China

**Keywords:** extraction, purification, structure, bioactivities, Eleutherococcus senticosus polysaccharides (ESPS, )

## Abstract

In recent years, natural polysaccharides have received growing attention and interest in view of their values in food, medical, cosmetics and other fields. *Eleutherococcus senticosus* (*E. senticosus*) is a medicine and food homologous plant that possess anti-tumor, anti-inflammatory, central nervous system and cardiovascular protection, anti-radiation, enhancement of human microcirculation, improvement of physical fatigue effects, mainly based on lignans, flavonoids and coumarin types. *E. senticosus* polysaccharides (ESPS), act as a kind of polysaccharide extracted and isolated from the root and rhizome of *E. senticosus*, have been found in many applications of medicine and food for their unique biological activity. Nevertheless, the existing studies are mostly concerned with small molecules of *E. senticosus*, less attention is paid to polysaccharides. Moreover, the types and structural characterization of ESPS reported in existing literature were also not summarized. In this paper, the research progress of ESPS is reviewed from the aspects of extraction, separation, structural characterization and biological activity, future perspectives from points of efficient extraction, resource utilization and quality control standards were also proposed, which provide reference for the further development and utilization of ESPS.

## Highlights

• The extraction and purification techniques of *Eleutherococcus senticosus* polysaccharides were summarized.
• Types and structural characterization of *Eleutherococcus senticosus* polysaccharides isolated and purified at present were preliminarily discussed.
• The modern pharmacological research and biological activity of *Eleutherococcus senticosus* polysaccharides were summarized, and its application prospect was proposed.

## Introduction

**FIGURE 1:**
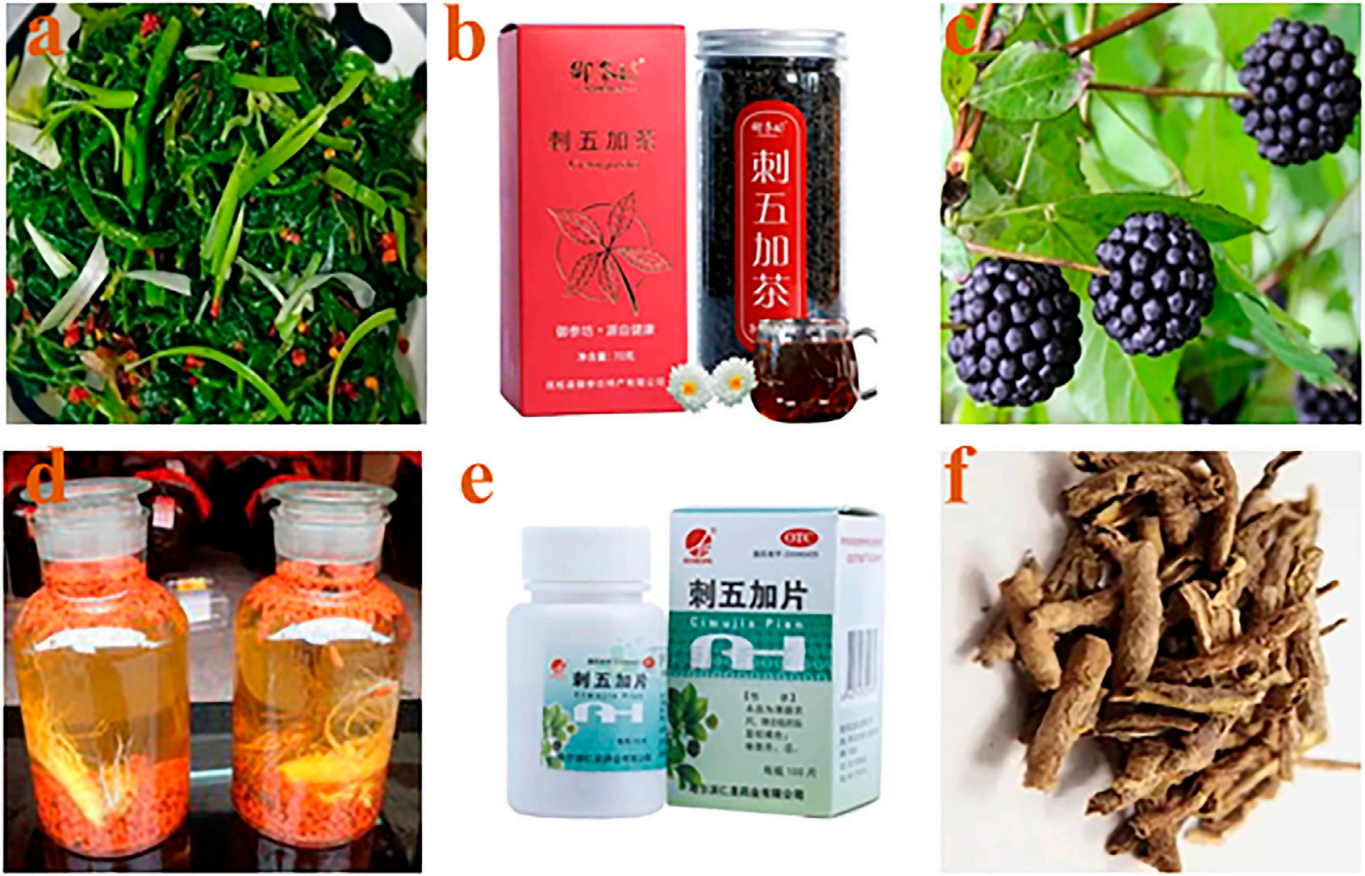
*Eleutherococcus senticosus.* Salad from leaves **(A)**; tea **(B)**; pulp **(C)**; wine **(D)**; tablets **(E)**; cortex **(F)**.

*Eleutherococcus senticosus*, commonly known as “Ci-wu-jia” or “*Acanthopanax senticosus*”, is botanically from the dried root and rhizome or stem of *Eleutherococcus senticosus* (Rupr. et Maxim.) Maxim [syn*. Acanthopanax senticosus* (Rupr. and Maxim.) Harms] belong to the Araliaceae. It is a medicine and food homologous plant that is widely distributed in northeast China, which contains multi-kinds of nutrients and components. Among variety of active ingredients, flavonoids (quercetin, hyperoside, quercetin, rutin, etc.), coumarins (isofraxidin, scopolamine, etc), lignans (ferulic acid glucoside, eleutheroside, carotin, syringin, etc.,) (Lee et al., 2012) played an important role in its activities. Moreover, some trace elements such as Ca, P, Mg, Fe, a variety of amino acids and polysaccharides also play a role in mediating the immune function of the body (Liu, 2010), antioxidant, anti-tumor, anti-inflammatory, central nervous system and cardiovascular protection, anti-radiation, enhancement of human microcirculation, improvement of physical fatigue, etc., (Luan, 2012; Pan, 2019a). As a medicinal and food homologous plant, various products such as oral liquid, tea, capsule preparation, wine of *E. senticosus* have been developed and commercialized, and favored by those who pay attention to health. The products of *E. senticosus* are listed in Figure 1.

**GRAPHICAL ABSTRACT:**
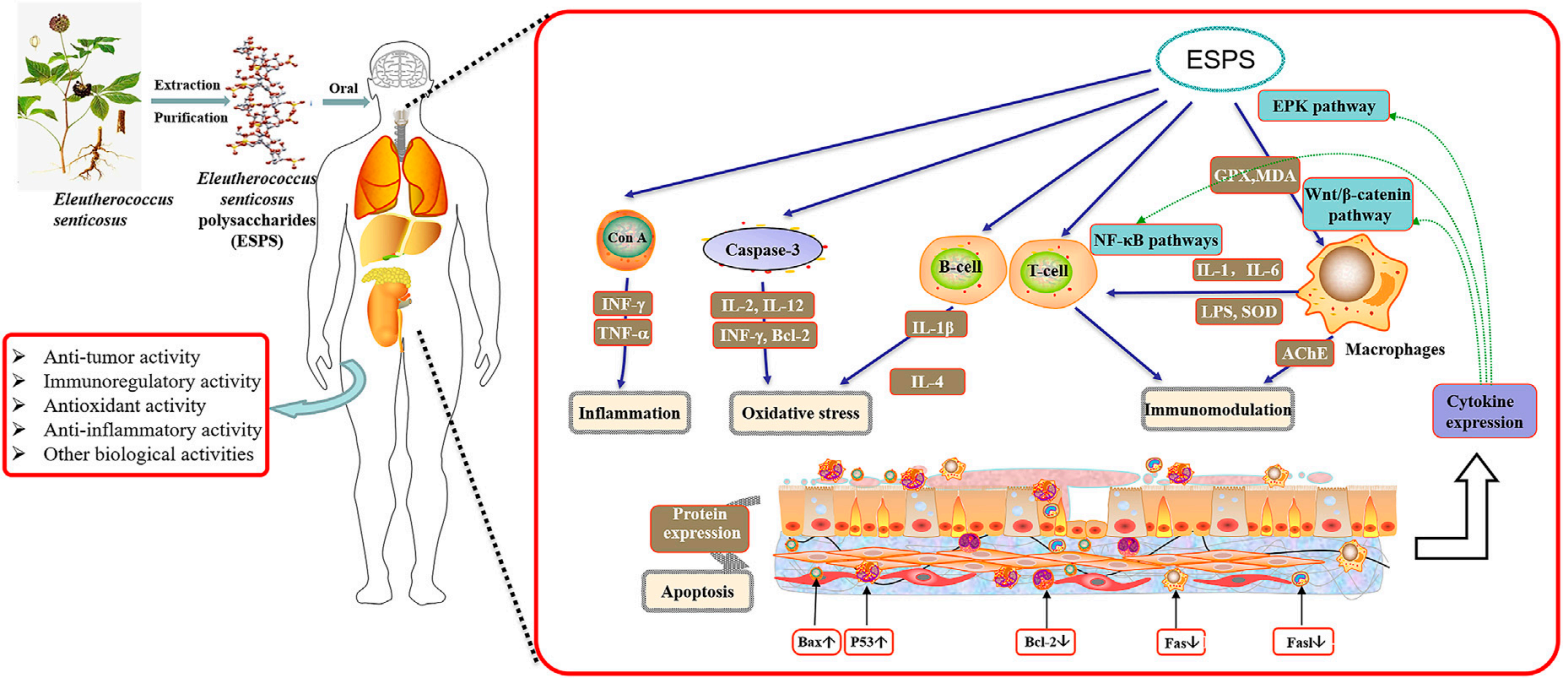


In recent years, polysaccharides have demonstrated good therapeutic effects against tumors, hyperlipemia, diabetes and hypertensive diseases (Shen et al., 1991; Duan, 2015). The development and utilization of polysaccharides have also become a hot spot. *E. senticosus* polysaccharides (ESPS) are an important biologically active ingredient extracted from the roots and rhizomes of *E. senticosus*. The content of alkaline polysaccharides in *E. senticosus* is about 2–8%, and that of the water-soluble polysaccharides is 2.3–5.7% (Gao, 2019). Studies had shown that ESPS possessed immunoenhancement (Xu et al., 2005), anti-tumor (Hao and Nan, 2013), anti-virus (Zhou, 2018), anti-fatigue, anti-oxidation, anti-diabetes (Fu et al., 2012), hepatoprotective, and other biological activities (Chen and Huang, 2018; Ganesan and Xu, 2019; Zeng et al., 2019), which prompts the huge application and development potential of ESPS. In this paper, the extraction and purification process and structure characterization of ESPS were systematically introduced, and the studies on biological activities of ESPS were further reviewed in order to expand the application of ESPS.

## Extraction and Purification

### Extraction of Crude Polysaccharides

#### Hot Water Extraction

Polysaccharides are a category of substances with higher polarity, higher water soluble and insoluble in EtOH. Because of the nature of polysaccharides, hot water extraction in combination with EtOH-precipitated has been commonly used in laboratories and industrial production. EtOH-precipitated polysaccharides are suitable for almost all water-soluble polysaccharides. The principal principle is to separate the polysaccharides by reducing the dielectric constant of the aqueous solution to dehydrate the polysaccharides for precipitation. There are several ways to extract ESPS (Table 1). Feng et al. used a single factor test to optimize the extraction conditions of ESPS and obtained the optimal condition: pulverization degree above 80 mesh, solute extraction 50% EtOH, extraction pH = 6, soaking for 2 h, degreasing treatment with petroleum ether. Under these conditions, the extraction yield of crude ESPS could reach 3.27 ± 0.07% (Feng, 2016). An orthogonal experiment was developed from the experimental data in order to predict the ESPS yield by Cheng, the optimal condition was found to be: extraction temperature 80°C, extraction once, time of 150 min and solid-liquid ratio of 1:20 g/ml with a maximum pectin yield (23.3 mg g^−1^) (Chen, 2011a). Based on the single factor test, some researches adopted the Box-Behnken center combination design test and response surface method (RSM) to compare rhizomes, fruits, and leaves, and finally determined that the fruit was the best extraction part. This technique gives a maximal yield of 13.21% for material-liquid ratio of 31:1 in 116 min, extraction temperature 95°C (Shen, 2017). Some scholars also investigated the drying method and found that the freeze-drying method was better than the spray-drying method (Zhang and Sun, 2005).

**TABLE 1:** Summary of the extraction process of ESPS.

Method	Pros	Cons	Optimization Factors	Optimization Results	ESPS Content	References
Hot water extraction	The production cycle is short, the experiment cost is low, experiment operation is simple and the degree of damage to the structure of the extract is small.	Part of the protein will be extracted and protein must be removed; low selectivity, low extraction rate.	Material/liquid, extraction time, times, temperature, alcohol precipitation temperature, time, pH	90°C, 150 min, material/liquid 1:18, extraction twice; alcohol precipitation condition -20°C, 36 h, ethanol extractant ratio 4:1, pH=7	263.3 mg/ml	Zhai (2007)
—	—	—	Extraction temperature, time, material/liquid l, times	80°C, 150 min, material/liquid 1:20, extract once extraction time> extraction temperature> extraction times> material/liquid	23.21 mg/g	Chen (2011a)
—	—	—	Material/liquid, extraction time, temperature, times	90°C, 2.5 h, material/liquid 1:25 (g/mL), extract 3 times.	23.95 mg/g	Chen (2015a)
—	—	—	*E. senticosus* pulverization degree, extraction solute, pH, soaking time degreasing solvent	degree of pulverization:>80 meshes, solute is extracted by 50% ethanol, pH = 6 (petroleum ether degreasing treatment soaking 2 h	3.27±0.07% (*n* = 3)	Feng (2016)
—	—	—	Extraction parts (rhizome, leaves, fruits) extraction time, temperature, alcohol concentration, material/liquid	95°C, 116 min, material/liquid 31:1 best extraction part fruit, alcohol concentration 85%,	13.21%	Shen (2017)
—	—	—	Extraction temperature, time, material / liquid	80°C, 1.5 h, material/liquid 1:21	10.14%	Yu (2019)
Ultrasonic extraction	More efficient, faster, high extraction yield, low energy consumption	Ultrasound will damage the structure of polysaccharides, resulting in difficulty in separation and difficult to apply to industrial production	Extraction temperature, material/liquid, time, ultrasonic power	58°C, 73 min, material/liquid 1:25; ultrasonic power 85 W	1.532±0.037% (*n* = 3)	Chen et al. (2018)
			Ultrasonic power, material/liquid , extraction temperature, time	57°C, 42 min, material/liquid 1:40 g/mL ultrasonic power 90 W	3.86%	Chen (2015b)
Microwave extraction	Strong penetration, uniform and fast heating, high selectivity.	Polysaccharides often contain some pigments, so they need to be decolorized.	—	It was the first time to extract ESPS using microwave technology and determine its content.	5.01%	Li and Liu (2003)
—	—	—	Extraction time, microwave power, material/liquid	22.5 min, material/liquid 1:25 22.5 min, material/liquid 1:25	1.52% ± 0.09% (*n* = 3)	Zhang and Guo (2015)
Alkaline extraction	Higher total glucose content	The yield of polysaccharide was slightly lower, and it will damage the structure of acid polysaccharide and neutral polysaccharide	Alkali concentration, material/alkali ratio, alkali extraction time	Alkali extraction time>material-alkali ratio>alkali concentration 10 h, material/alkali 1:16. alkali concentration 4%,	5.70%	Wang (2006)
—	—	—	Graded alcohol precipitation, step-by-step alcohol precipitation	Used step-by-step alcohol precipitation method, more and higher content of polysaccharides can be obtained	6.01%	Li (2019)

However, the hot water extraction method will introduce some impurities like protein, which needs to be removed. Sevag reagent, trifluoro trichloroethane, trichloroacetic acid, anion and cation exchange resin and the enzymatic hydrolysis are the commonly used mediums. In ESPS literature, protein is mostly removed by the Sevag method (Jiao, 2008; Li, 2019). Wang et al. compared the deproteinization effects of Sevag, trichloroacetic acid (TCA)-Sevag, chitosan coagulation and enzyme on ESPS. The results showed that the protein removal rate: enzyme 93.1% = chitosan coagulation 93.1% > TCA-Sevag 83% > Sevag 28%, polysaccharide loss rate: chitosan coagulation 47% > TCA-Sevag 43% > Sevag 18% > enzyme 7%. Results indicated that enzyme deproteinization has the best effect (Wang, 2009).

#### Ultrasound-Assisted Extraction (UAE)

The ultrasound-assisted extraction (UAE) method were employed for increasing the movement frequency and speed of macromolecules, strengthening the solvent penetration ability, and shortening the extraction period of polysaccharides by ultrasonic waves (Cui, 2012). In some experiments, the RSM was used to optimize the extraction of ESPS by ultrasound-assisted extraction (UAE), by which the optimum levels of the parameters were obtained as follows: extraction temperature 58°C, extraction time 73 min, liquid/solid ratio 25:1, ultrasonic power 85 W (Chen et al., 2018). However, the extraction time should not be too long because ultrasound has a mechanical shearing effect, which may damage the structure of ESPS. Moreover, the UAE method is difficult to be widely applied in the field of industrial production in view of the limitations of the instruments.

#### Microwave Extraction

Water molecules in the cell are rapidly heated and pressurized under microwave due to its strong penetrating power, which increased the extraction rate by breakage of cell wall and release of intracellular polysaccharides into the extract.

Li et al. removed fat-soluble impurities and interfering components gradually with petroleum ether, ether and EtOH and then ectracted *E. senticosus* crude polysaccharide by microwave technology (Li and Liu, 2003). Zhang et al. used the RSM based on Box-Behnken design to optimize the extraction of ESPS from three aspects of the microwave power, microwave processing time and material-to-liquid ratio parameters. The results showed that microwave extraction 22.5 min, microwave power 450 W, material-liquid ratio 1:25 (g·ml^−1^) achieved the best performance (Zhang and Guo, 2015). Furthermore, microwave extraction of ESPS often requires depigmentation. Ion exchange, adsorption, oxidation and metal complexation are common methods for subsequent depigmentation.

#### Alkali-Extraction

Polysaccharides could be divided into acidic, alkaline and neutral polysaccharides, which can be extracted by adjusting the acidity and alkalinity like some small molecule compounds. For example, Jiang et al. extracted lemon polysaccharides under alkaline conditions with an extraction rate of 8.81% (Jiang et al., 2017), Li et al. used alkaline leaching method to optimize the extraction of acidic Tuckahoe polysaccharides, the extraction rate was increased to 78.5% (li, 2018). Similarly, corn silk polysaccharide was comparatively extracted by acid extraction and water under the same experimental conditions. The extraction rate 33.36% obtained by acid was higher than the water extraction (7.44%) (Li and Zhou, 2020). Thus, polysaccharides could also be extracted by the acid and alkaline leaching method due to their hydrolysis. However, little related literature on extracting ESPS were obtained in view of the fact that the acid-base method may destroy the structure of the active polysaccharides.

Li et al. compared the extraction of ESPS with water extraction and alkali-extraction. The yield of crude polysaccharides from water extraction and EtOH precipitation was 20.8%, and total carbohydrate content in which was 5.19%. Alternatively, the value obtained from alkali-extraction and EtOH precipitation were 18.31 and 6.01%, respectively (Li, 2019).

#### Other Methods

**FIGURE 2:**
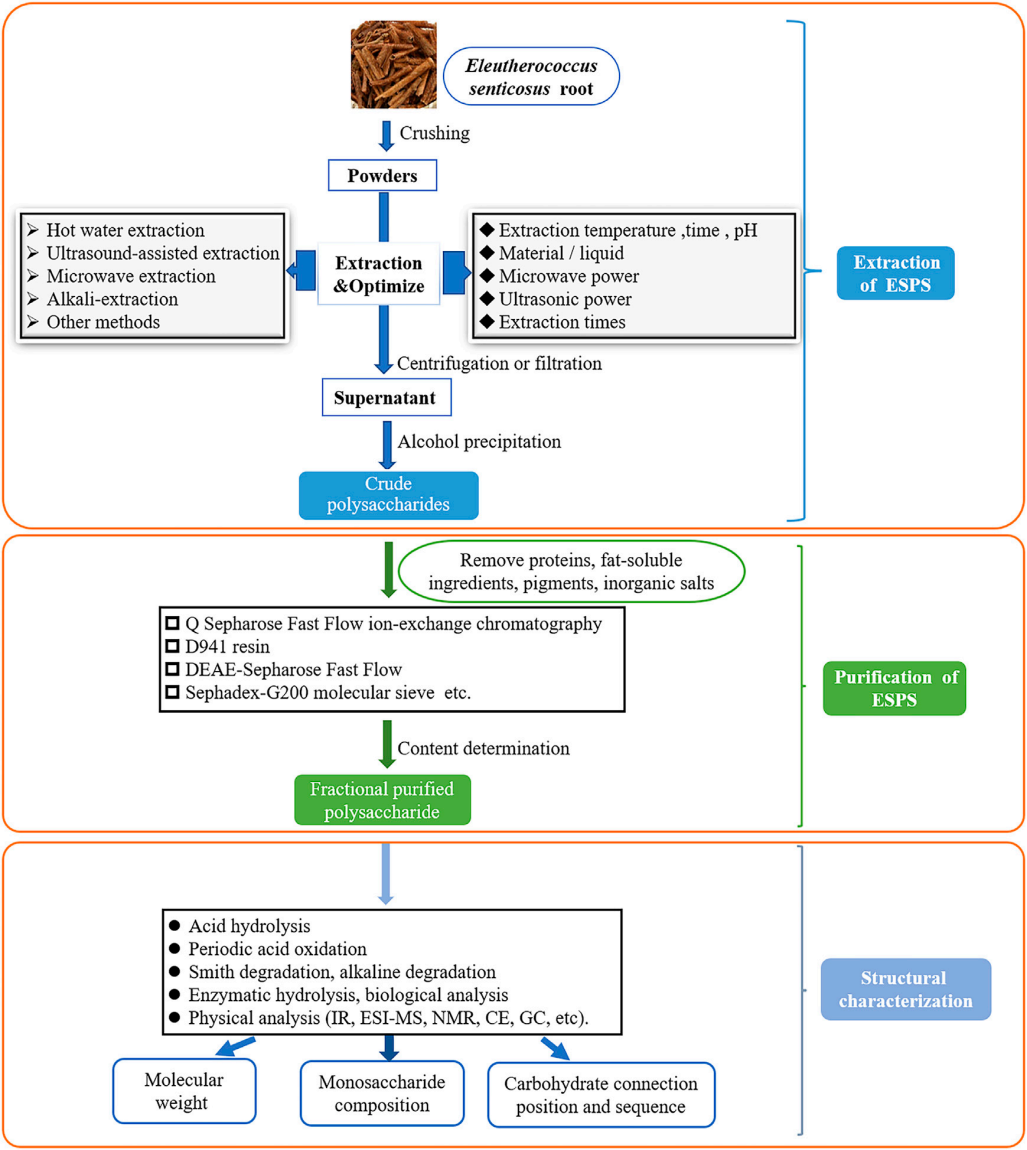
Schematic diagram of extraction, purification and structural characterization of ESPS.

Aside from the common technologies mentioned above, enzymatic hydrolysis, supercritical fluid extraction (SFE) (Wei, 2018), enzyme-microwave-ultrasonic-assisted extraction (Yin et al., 2018) were also employed in the extraction of plant polysaccharides. However, these methods have not been applied to the ESPS extraction at present, which needs further research. Moreover, the crude polysaccharides extracted by above methods need to remove proteins, fat-soluble ingredients, pigments, inorganic salts and other small molecule impurities before subsequent tests. Figure 2 summarized the diagram of extraction, purification and structural characterization of ESPS.

### Purification of Eleutherococcus Senticosus Polysaccharides

Polysaccharides in medicinal materials often coexist with other active ingredients. The separation and purification of homogeneous polysaccharide from crude polysaccharides was usually achieved by EtOH precipitation according to the solubility differences in EtOH. Normally, EtOH precipitation could be divided into step-alcohol-sinking and graded-alcohol-sinking, respectively. Related experiments showed that the purification performance of ESPS by step-alcohol-sinking performed more perfect in view of the higher content (Li, 2019). Besides, the concentration of alcohol in the graded-alcohol-sinking method would also affect the biological activity of ESPS. Li et al. compared the immune enhancement effects of ESPS obtained from 20, 40, and 60% alcohol precipitations. After comparison, ESPS obtained from 40% EtOH-precipitated exhibited better resistance to the splenic lymphocyte proliferation induced by ConA and LPS in a dose-dependent manner (Li, 2020).

In recent years, new materials and technologies have been increasingly applied in the separation and purification of polysaccharides accompanied by the rapid development of the science and technology. Among which, ion exchange chromatography has been used for the preparation of various types of components of traditional Chinese medicine in view of their advantages of good separation effect, fast separation speed, wide application range, and simple operation. Usually, diethylaminoethyl (DEAE)-cellulose is first used as an exchange agent to purify the polysaccharide to be acidic or neutral. The separated one was further purified by Sephadex gel according to the difference in molecular size. Based on this principle, scholars have developed different classification and types of fillers to achieve the systematic separation of polysaccharides with different properties. For example, Wang et al. employed Q Sepharose Fast Flow ion-exchange chromatography column to purify three kinds of ESPS,NASC-1, ASH-1 and ASA-1 that extracted by cold alkali, hot alkali and acid solutions, respectively (Wang, 2006). Bai et al. classified the ESPS from the rhizomes of *E. senticosus* into neutral ASPN and acidic ASPA-1 and ASPA-2 by DEAE-cellulose column. Subsequently, Sepharose CL-6B was further used to separate ASPA-1 into two uniform grades, namely ASPA-1-A and ASPA-1-B (Bai, 2015). Similarly, Zhai et al. adopted D941 resin, DEAE-Sepharose Fast Flow to chromatograph the crude polysaccharide and then used Sephadex-G200 molecular sieve to obtain the purified neutral and acidic polysaccharide As-1 and As-2, respectively (Zhai, 2007).

### Content Determination

Content determination is an important procedure to control and evaluate the quality of polysaccharide products. Phenol-sulfuric acid and anthrone-sulfuric acid methods were the most commonly used ones. The principle is that polysaccharides are hydrolyzed into monosaccharides and further dehydrated to furfural or carboxymethyl furfural due to the concentrated sulfuric acid. Subsequently, phenol or anthraquinone can undergo condensation reaction with furfural derivatives to produce orange-yellow and blue-green compounds. Within 100–200 mg, the color depth increases with the increase of polysaccharide content, which can be determined by colorimetry. The compound produced by the phenol-sulfuric acid method has a maximum absorption peak at a wavelength of 490 nm, so the absorbance of the sample is measured at λ = 490 nm (Bai, 2015).

A study compared the phenol-sulfuric acid and the anthrone-sulfuric acid methods for the determination of ESPS content and found that the results of the two methods were similar, but the phenol-sulfuric acid method was more convenient. Using phenol-sulfuric acid method, the content of ESPS after freeze-drying was 51.9–56.5%, while that obtained by spray-drying was 50.5–53.1% (Zhang and Sun, 2005).

Existing data from literatures reveal that the variation explained by the content of ESPS ranged from 1.52 to 13.21% due to the limitation of the extracted part and process of *E. senticosus*. How to further increase the content of ESPS is still the direction we need to work hard on.

### Structural Characterization of Polysaccharides

The polysaccharide belongs to a kind of high molecular polymer with a relative molecular weight (Mw) ranging from tens of thousands to tens of millions connected by glycosidic bonds. The structure identification of polysaccharides could be accomplished by the determination of the purity, monosaccharide composition, Mw, position and sequence of carbohydrate connection, glycoside bond configuration and oxygen ring. Several technologies including acid hydrolysis, methylation, periodic acid oxidation, Smith degradation, alkaline degradation, enzymatic hydrolysis, biological analysis, physical analysis (including IR, MS, ESI-MS, MALDI-MS, NMR, CE, GC, etc.,) would be employed.

The early research on structure of ESPS was limited to incomplete and inaccurate characterization by equipment and technology. Mw and monosaccharide composition were the only detected items for ESPS. The Mw of polysaccharides was obtained by measuring the standard dextran with known Mw. Based on this approach, PES-A and PES-B with 7 and 76 kDa together with immunologically active polysaccharides As-II and As-II with 150 and 30 kDa were purified from the root of *E. senticosus* (Xu, 1983)*.* In the 21st century, the structure research and chemical modification technology of ESPS become more mature, high performance gel permeation chromatography (HPGPC) analysis technology with more accurate and fast superiority is developed to determine the Mw distribution and uniformity of polysaccharides based on the molecular sieve principle. Accordingly, several ESPS with Mw ranging from 7 to 400 kDa were reported (Wang, 2006). Their structural information is provided in Table 2. It is worth noting that the separation range of the HPGPC column possess the significant influence on the determination results. Bai et al. compared the Mw of the same ESPS samples separated by two specifications of columns from TSK-gel company. Results showed that Mw of ESPS-1-A and ASPA-1-B were 48 and 11 kDa by G-3000PWXL, while the value were 37 and 10 kDa by G-4000PWXL, respectively (Bai, 2015). As ASPS-1-A possessed larger Mw, more significant influence on it was present. This reminds us that the choice of chromatographic column is very important.

**TABLE 2:** **S**tructural characterization of polysaccharides isolated from the different parts of *Eleutherococcus senticosus.*

No	Compound Name	Medical Parts	Monosaccharide Composition^a^	Main chain composition/ structural domain	Branch composition	M.W. (KDa)	Reference
1	PES-A	Root	Glc: Gal: Ara =3.3:2:1	—	—	7	Xu (1983)
—	PES-B	Root	—	—	—	76	Xu (1983)
—	PES-W	Root	—	—	—	—	Xu (1983)
2	As-II	Root	Glc	—	—	150	wang (1986)
—	As-III	Root	Ara: Xyl:4-O-methyl-D-GlcA =1:11:1	—	—	30	wang (1986)
3	AS-2	Root	Ara: Xyl: Rha: Gal: Glc =1.6:1.2:1.8:1.0:3.6	β-(1→3)-Glc, β-(1→4)-Glc	(1→4)-Rha, (1→6)-Gal, (1→3)-Gal, (1→6)-Glc	78	Zhang et al. (1993)
4	ASP	Root	—	—	—	—	Han et al. (2003)
5	ASC-1	Root	—	—	—	—	Wang et al. (2006)
—	ASC-2	Root	Rha: Ara: Xyl: Glc: Gal	—	—	—	Wang et al. (2006)
—	ASC-2C	Root	—	—	—	17	Wang et al. (2006)
—	ASC-2S	Root	—	—	—	11	Wang et al. (2006)
6	ASC-1P2B	Root	Xyl	—	—	—	Wang et al. (2006)
7	ASH-1	Root	—	—	—	—	Wang et al. (2006)
—	ASH-1P3	Root	Rha: Ara: Xyl: Man: Glc: Gal	—	—	—	Wang et al. (2006)
8	ASA-1	Root	—	—	—	12	Wang et al. (2006)
—	ASA-2	Root	—	—	—	—	Wang et al. (2006)
9	ASA-1P3A	Root	Glc: Xyl: Gal= 22.4:5.2:1	(1→6)-Glc	(1→4)-Glc, (1→3)/(1→4)-Xyl,	38.3	Wang et al. (2006)
—	ASA-1P3B	Root	Glc: Xyl: Gal =7.6:19.4:1	(1→2)-Glc , (1→4)-Xyl	(1→3)-Xyl, (1→2), (1→4), (1→6)-Glc	7.2	Wang et al. (2006)
10	ASP-2-1	Leaf	Rha: Xyl: Glc: Man: Ara: Gal: GlcA =7.45:18.63:25.15:0.93:8.35:2.79:5.69	—	—	14.6	Chen (2011b)
11	ESPS	Root	Man: Rha: GlcA: GalA: Glc: Gal: Xyl: Ara: Fuc=1.5:13.6:0.6:14.9:17.1:24.2:11.6:15.4:1.1	—	—	17	Bai (2015)
—	—	—	GlcA: Rha: Xyl: Fru: Glc=1.10:6.38:2.30:2.83:12.34	—	—	—	Zhang and Sun, (2015)
—	—	—	Ara: Glu: Gal: Xyl: GalA =51.4:24.5:10.2:5.7:4.9	—	—	74,38,45,23	Wang et al. (2016)
—	—	—	Ara: Xyl: Gluc: Man=7.1:22.3:7.6:1.0	—	—	—	Meng (2018)
12	ASPA	Root	Man: Rha: GlcA: GalA: Glc: Gal: Xyl: Ara: Fuc= 0.3:13.5:1.9:27:3.6:22.3:15.1:15.7:0.6	RG-1, AG-II, XGA	—	—	Bai (2015)
13	ASPN	Root	Man: Rha: GlcA: GalA: Glc: Gal: Xyl: Ara: Fuc =4.3:4.5:1:1.7:25.7:32.9:7.1:22.6:0.2	—	—	—	Bai (2015)
14	ASPA-1	Root	Man: Rha: GlcA: GalA: Glc: Gal: Xyl: Ara = 0.2:13:0.8:35.9:0.6:17:25.3:7.2	RG-1, XGA	—	—	Bai (2015)
—	ASPA-2	Root	Man: Rha: GlcA: GalA: Glc: Gal: Ara = 0.4:11.4:1.8:15.7:2.2:44.7:23.8	RG-1	—	400	Bai (2015)
15	ASPA-1-A	Root	Man: Rha: GlcA: GalA: Glc: Gal: Xyl: Ara: Fuc=0.4:14.8:1.1:26.9:0.6:20.4:27.1:8.3:0.4	RG-1, XGA	—	48,40,37	Bai (2015)
—	ASPA-1-B	Root	Man: Rha: GlcA: GalA: Glc: Gal: Xyl: Ara: Fuc=0.4:11.7:0.9:46.2:1:14.1:20.4:5.1:0.2	RG-1, XGA	—	11,10	Bai (2015)
16	ABPS-21	Stem	Gal: Glc: Rha: GalA =3.0:2.0:2.0:1.0 →4)-D-Galp-(1→4)-D-Glcp-(1→4)-D-	α-L-Rhap-(1→4)-β-D-Glcp-(1→,	106	—	Hu et al. (2015)
—	—	—	—	Galp-(1→4)-D-Galp-(1→2)-α-L-Rhap-(1→,	α-D-GalpA-(1→O-6 1,4,6-linked β-D-Glcp	—	—
—	—	—	—	—	α-L-Rhap-(1→4)-β-D-Glcp-(1→,	—	—
—	—	—	—	—	α-D-GalpA-(1→ O-6 1,4,6-linked β-D-Galp	—	—
—	ABPS-21P	Stem	Gal: Glc: Rha =3.0:1.0:1.0	—	—	8.25	Hu et al. (2015)
17	ANP	Buds	Ara: Man: Glc: Gal=1.0:2.6:2.5:1.4	—	—	10.7	Lee et al. (2015)
—	AAP	Buds	Ara: Gal: 4-O-methyl-D-GlcA = 5:10:1	AG-II	—	84	Lee et al. (2015)
18	CASPs	Root	Ara: Man: Rha: Gal: Glc =1:1.1:3:4.7:5	—	—	70,38,120,19	Xie et al. (2015)

a Ara, arabinose; Fuc, fucose; Fru, fructose; Gal, galactose; Glc, glucose; GalA, galacturonic acid; GlcA, glucuronic acid; Man, mannose; Rha, rhamnose; Rib, ribose; Xyl, xylose; AG, arabinogalactans; RG, rhamnogalacturonan; XGA, xylogalacturonan.

The determination of monosaccharide composition of polysaccharides has been improved and perfected. In the initial stages, thin layer chromatography (TLC) method was used by comparing the specific Rf value with each monosaccharide standard. While TLC can only provide the type of monosaccharides like ASC-2 and ASH-1P3 (Wang, 2006), their proportion could not be given as TLC is an unquantifiable method. Instead, gas chromatography (GC) could realize the measurement of above two respects based on the derivatization method. After GC analysis, the proportion of monosaccharides of PES-A (Xu, 1983), ASA-1P3A and ASA-1P3B had been supplied, which also provides key information for the subsequent estimate of ESPS types. Besides, the results in Table 2 showed that GalA and Gal possessed relatively high proportion in ESPS.

In recent years, more in-depth research into chain sequencing and structural domain of carbohydrate is developed based on the requirement of pharmacology, toxicology and structural modification. According to the sequence, mode, and monosaccharide types of glycosylation in the main chain, the structure of ESPS could be divided into three types, namely RG-I, XGA and AG-II. 1) RG-I: Most of ESPS belong to the polysaccharides with RG-I structure due to high content of Rha and GalA connected through 1→4 linkage. Besides, the Rha/GalA ratios in different samples ranged from 0.25 to 0.73 indicated that they may be inducted as pectin. 2) XGA: XGA domain represent for some polysaccharides with higher Xyl content. Accordingly, the Xyl/GalA ratios 1.0 and 0.44 obtained from ASPA-1-A and ASPS-1-B suggest that XGA domain existed in both components. Moreover, several XGA domains also coexist with RG-I domain in other ESPS samples (Bai, 2015). 3) AG-II: Three kinds of ESPS named ASPA (Bai, 2015), ANP (Lee et al., 2015), and AAP (Lee et al., 2015)were found as AG-II pectin due to major connection of Ara and Gal through 1→3 linkage. However, the structure of ESPS was irregularly in branch composition.

## Biological Activities

### Anti-tumor Activity

#### Anti-lung Cancer Activity

Lung cancer remains the leading cause of cancer deaths all over the word. It is generally divided into non-small cell lung cancer (NSCLC) and small cell lung cancer (SCLC), respectively. However, SCLC was harder to cure due to its high invasiveness, low tumor differentiation, high deterioration as well as poor results of chemotherapy and surgery. Some studies had confirmed that ESPS could inhibit the proliferation of human SCLC cell line H446 using MTT, FCM and Hoechst33258 staining methods (Zhao, 2008a), and promote the apoptosis of H446 cells by up-regulating the expression of Bax and p53 genes and down-regulating the expression of Bcl-2 genes (Zhao, 2008b), and lead to G2/M phase arrest to interfere with H446 cell cycle progression by activating the ERK1/2 pathway (Zhao, 2010). Besides, the intervention effect of ESPS on the incidence of Lewis LC was also observed. *In vivo*, inhibition of tumor growth by ESPS was also manifested by decreasing the levels of PAI-1 and μPA in plasma, tumor and lung tissue. Moreover, combined administration of cyclophosphamide (CTX) and ESPS exhibited better effect than a single group of drugs (Zhang, 2001). Similarly, Sun et al. explored transwell and wound healing assay to evaluate the anti-tumor activity of ESPS on NCI-H520 cells after 24 and 48 h, decreased proliferation as well as repressed invasion and migration associated with Wnt/β-catenin pathway mediated-EMT provided new theoretical knowledge for NSCLC (Sun et al., 2019). In addition, Aidi injection is a proprietary Chinese medicine extracted from *E. senticosus* to treat stage IIIB/IV non-small cell lung cancer, and this therapeutic effect seems to be related to ESPS (Wang et al., 2018).

#### Anti-liver Cancer Activity

In recent years, some studies have found that ESPS also showed remarkable biological activity against liver cancer. In 1997, Zhang et al. found that ESPS had a significant inhibitory effect on hepatocellular carcinoma induced by chemical carcinogens (3'-me-DAB) (Zhang et al., 1997). *In vitro* experiments showed that ESPS could induce apoptosis and G0/G1 phase cell cycle growth arrest in HepG2 cells by targeting Wnt/β-catenin pathway (Wang et al., 2016). Anti-hepatic carcinoma assay experiment of ASC-1 and ESPS on mouse H22 transplanted tumors also showed a preventive effect. Compared with the white water group, the tumor suppression rate of ASC-1 reached 39.13% (Wang, 2006), and the tumor quality of the ESPS group was significantly lower than that of the control group (Meng, 2018). It can be inferred that ESPS indirectly suppressed hepatic carcinoma by enhancing the immunity of the body.

#### Other Anti-cancer Activity

Malignant tumors remain a serious disease that threatens human health. Currently, previously discovered ESPS have shown effects on cervical cancer, sarcoma, and laryngeal cancer of varying degrees along with the advantages of safety, efficacy and low toxicity. It can be used as antineoplastic and anti-tumor adjuvant drugs. As early as 1993, Cao et al. found that intragastric administration of ESPS at 10 mg/kg promoted the proliferation of mouse spleen cells and enhanced the activity of LAK cells, which provides an ideal biological response adjustment for LAK/IL-2 tumor cell therapy agents (Cao and Du, 1993). Meng et al. founded that different doses of ESPS (200, 100 and 50 mg/kg) could significantly inhibit the growth of sarcoma S180 and cervical cancer U14 on tumor-bearing mice and prolonged their survival time. Among which, 100 mg/ml dose possessed the best activity (Meng, 2018). Simultaneously, Tong et al. confirmed that ESPS has a direct anti-tumor effect on sarcoma S180 cells, whose inhibition rate exceeds 70% and half effective inhibitory concentration is 0.38 g/L (Tong, 1994). Besides, studies have also shown that ESPS can inhibit the proliferation of human cervical cancer HeLa cells via promoting the expression of Bax protein. At the same time, it may down-regulate the expression of survivin protein (Jin, 2014; Jin, 2016), thereby promoting the apoptosis of HeLa cells. ESPS inhibited the proliferation of CD133-positive stem cells in laryngeal cancer Hep-2, which may be related to the influence of ESPS on the expression levels of PD-L1 and Bcl-2 to promote stem cell apoptosis (Chen et al., 2019). This important discovery lays the foundation for the targeted therapy of laryngeal cancer stem cells.

### Immunoregulatory Activity

Immunomodulation is considered to be one of the important physiological functions for the body to identify and remove antigenic foreign bodies as well as maintain physiological dynamic balance and relative stability. Abnormalities in the immune system could cause a variety of diseases such as infection, inflammation and cancer (Yu et al., 2018). In recent years, ESPS has been proved to have a certain regulatory effect on the immune system of mice. On the one hand, it can directly activate the immune function of T, B lymphocytes and macrophages by regulating immune organs; on the other hand, it can also play its biological activity by regulating the level of cytokines. Many studies have shown that intraperitoneal injection or intragastric administration of ESPS could increase the weight of thymus and spleen and improve immune function in mice (Chen, 1984; Xie, 1989a; Xu, 1990). Moreover, this regulatory effect may be positively correlated with the dose. Sun et al. found that the immune organ index, peripheral blood leukocyte count and macrophage phagocytosis of CTX-induced immunocompromised mice were increased by intragastric administration of different doses of ESPS aqueous solution (6 mg/10 g, 12 mg/10 g and 24 mg/10 g), and the efficacy of high dose group was significantly (Sun et al., 2018). Equally, Luo et al. also found that high-dose ESPS could significantly promote the proliferation of spleen lymphocytes and increase the ratio of CD3^+^CD4^+^/CD3^+^CD8^+^ (Luo, 2013a). In addition, ESPS can also improve the innate immune function of the body by reducing the levels of cytokines such as IFN-γ, IL-2, IL-6 and TNF-α (Diao, 2008a; Han et al., 2014; Zhang, 2015). *In vitro*, ESPS could significantly promote lymphocyte proliferation induced by concavalinA and LPS, and this effect is not related to endotoxin (Chen et al., 2011). These studies provide abundant evidence for ESPS as a natural immunostimulant agent. However, the present research on the immune activity of ESPS remains on the animal level, and whether it can be used in clinic needs more in-depth research.

### Antioxidant Activity

As we all know, the production of free radicals has the beneficial effects of regulating cell growth and inhibiting viruses and bacteria (Diao, 2008c). Nevertheless, excessive free radicals will lead to excessive reactive oxygen species (ROS) formation and break the redox homeostasis. This is closely related to some human chronic diseases, such as cancer, arteriosclerosis and aging (Diao et al., 2010b). ESPS have been proved to have significant scavenging effects on a variety of free radicals, such as hydroxyl radicals, superoxide anions and hydrogen peroxide. *In vitro*, Chen and Zhao reported that ESPS had obvious scavenging activity evaluated by ferric reducing antioxidant power assay (FRAP), Fenton’s reaction, pyrogallol’s auto oxidation and DPPH radical scavenging experiments in a concentration-dependent manner (Jiao, 2008) (Meng et al., 2005; Diao, 2008d; Chen, 2011b). Xia et al. compared the antioxidant capacity of two water-soluble polysaccharides (ASP-B2 and ASP-B3) extracted from the leaves of *E. senticosus.* It is worth noting that the clearance rates of ASP-B2 and ASP-B3 for DPPH reached 91.75 ± 0.59% and 91.58 ± 1.58% at 2.0 mg/ml, respectively (Diao, 2008b). Likewise, ESPS extracted from fruits also showed antioxidant activity. Among the concentration range from 0.05 to 0.8 mg/ml, the scavenging rates of ESPS to DPPH and ABTS increased in a concentration-dependent manner, and the highest scavenging rates were 65.21 ± 1.85% and 58.02 ± 1.25%, respectively (Diao, 2009b).

Meantime, researchers also focused on the antioxidant activity of ESPS *in vivo.* It is reported that the activities of superoxide dismutase (SOD) and GSH-Px increased in a dose-dependent manner after 15 days of intragastric administration of ESPS (50, 100 and 200 mg/kg/d) in rats with cerebral ischemia-reperfusion. As SOD is the most important antioxidant enzyme for scavenging free radicals *in vivo*, the result suggests that ESPS may improve cerebral ischemia-reperfusion injury by improving the antioxidant capacity of brain tissue (Xie et al., 2015). Similarly, Long et al. found that ESPS could also improve the growth performance of chicks by increasing the activities of SOD and GSH-Px (Long et al., 2021). Besides that, some domestic scholars have also found that ESPS can significantly protect rat hippocampal neurons from oxidative stress damage induced by H_2_O_2_ (Diao et al., 2010b; Liu et al., 2013). Above studies indicated that ESPS seem to be used as a potential natural antioxidant. However, there is a big gap between the clinical application and fundamental research, which need further explore in the future.

### Anti-inflammatory Activity

Inflammation is a response to pathogens and tissue damage. This process is mainly caused by a series of pathological reactions of the injured site by stimulating immune cells to release inflammatory mediators. However, long-term inflammation is the main cause of aging and serious diseases, such as inflammatory bowel disease, cardiovascular disease (CVD), hepatitis and cancer (Diao, 2009d). In the past decades, the natural polysaccharides extracted from *E.* senticosus showed significant anti-inflammatory activity. Many studies have shown that ESPS could regulate the level of inflammatory mediators *in vivo* and reduce the infiltration of inflammatory cells in multiple organs. It is reported that ESPS pretreatment reduced the secretion and expression of inflammatory cytokines such as IL-2, IL-4 and INF-γ, which has a certain protective effect on immune liver injury (Zhu et al., 1982; Xie, 1989c; Luo, 2013b; Han, 2013; Zhang, 2018; Zhai, 2020). Similarly, ESPS could also inhibit the activation of NF-κβ in mouse liver tissue and endotoxic shock induced by LPS/D-GalN (Diao, 2009c). Xie et al. also reported that intragastric administration of ESPS could reduce the contents of IL-1β and TNF-α in brain tissue and exhibit a certain protective effect on cerebral ischemia-reperfusion injury (Zhang et al., 2019). In addition, Han and Fan et al. have verified that ESPS could improve a variety of inflammatory bowel diseases induced by LPS, whose effect is mainly mediated by NF-κB/MLCK, HIF-1α/COX-2 and TLR4/NF-κB signal pathways (Yang et al., 1983; Yang et al., 1985; Poolsup et al., 2004; Luo, 2008; Mo, 2012a; Mo, 2012b; Han et al., 2016a; Han et al., 2016b; Lu, 2016; Yang, 2016; Han et al., 2017; Pan, 2019b; Zhang et al., 2020a). Clinically, Poolsup et al. conducted a statistical analysis of 433 patients with upper respiratory tract infection and found that *E. senticosus* was more effective than placebo. Whether this effect is related to ESPS needs to be further explored (Poolsup et al., 2004).

### Other Biological Activities

Apart from to above biological activities, ESPS also possess the activities of analgesia, anti-radiation, anti-fatigue as well as compatibility with other drugs to treat diabetes and leukemia. As early as the end of the 20th century, ESPS was reported to increase the ability to produce IFNs in S801 and S7811 leukemia cell lines (Diao, 2008b; Diao, 2008c; Diao, 2008d; Diao, 2009a; Diao, 2009b). Similarly, the proliferation of leukemia cells K562 and Pmur388 original from human and mice were also inhibited by ESPS (Diao, 2009c; Jin, 2016). Moreover, Mo et al. reported that ESPS also had anti-radiation effect (Diao et al., 2010a; Diao et al., 2010b). ESPS has also shown a protective effect on the gastrointestinal tract, reproductive system, and peripheral blood cells of rats irradiated by 60Co gamma rays (Diao et al., 2010c). It’s worth noting that magical effect of ESPS as an adjuvant therapy for diabetes was discovered by the rapidly reduced blood glucose after combined use of ESPS with Metformin, whose efficacy on the level of blood lipids (TC and TG), thiobarbituric acid reactive substances (TBARS), AST, ALT, ALP, total bilirubin, creatinine and urea was better than that of Metformin (Fu et al., 2012).

**FIGURE 3:**
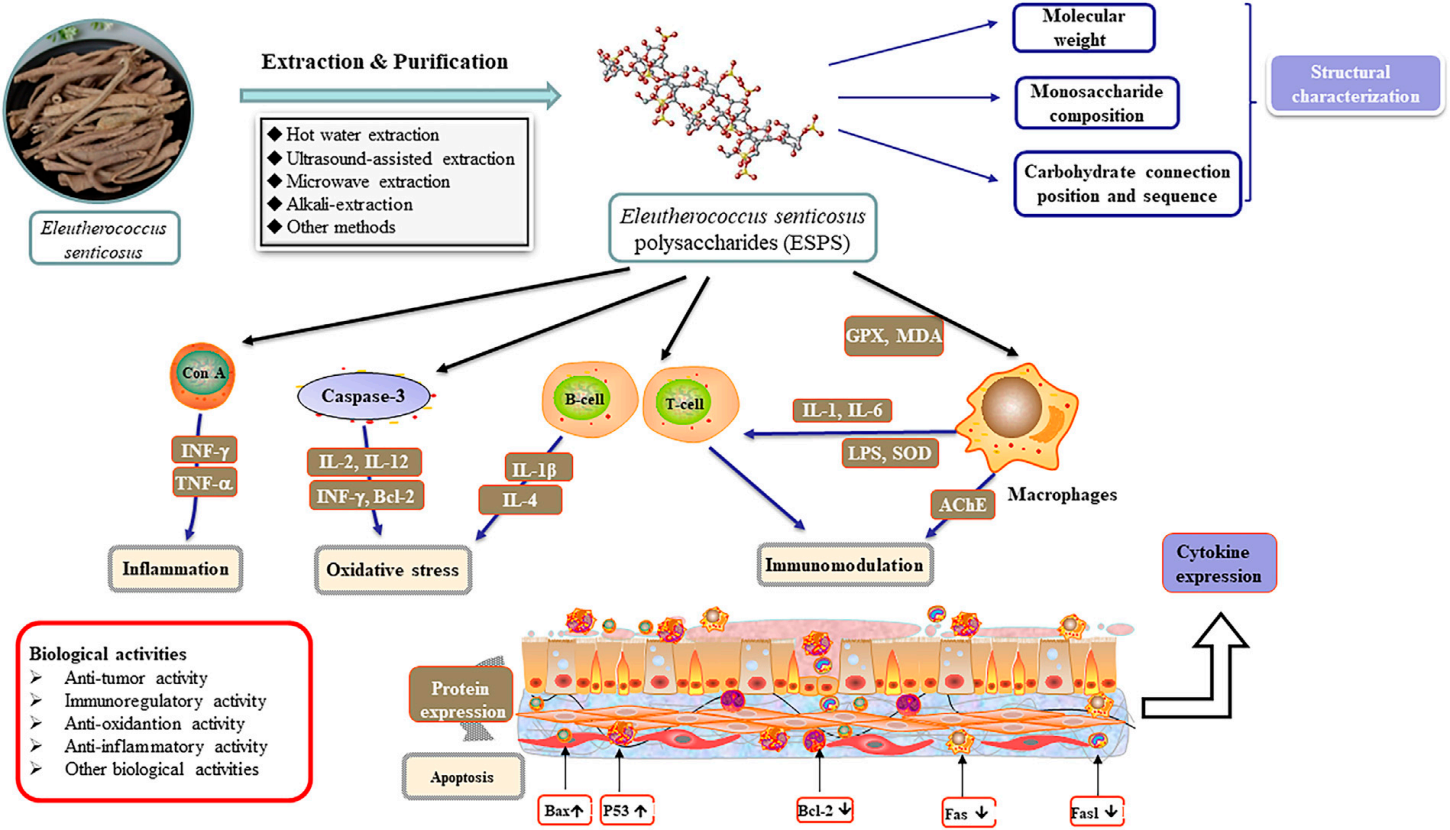
Schematic diagram of biological activity mechanism of ESPS.

Besides, ESPS can also improve exercise-induced energy metabolism, increase serum creatinine level and reduce protein decomposition, which exhibits a significant anti-fatigue potential (Han, 2007; Bang et al., 2015). Others used metabonomics methods to investigate the mechanism of ESPS on Alzheimer’s disease and CVD rats by detecting the endogenous substances, 20 potential biomarkers such as 6-dimethylaminopurine and l-acetylcarnitine were screened out (Lu, 2015). Table 3 and Figure 3 summarize the biological activities and related mechanism of ESPS, respectively.

**TABLE 3:** Pharmacological activities and involved mechanism of ESPS.

Biological activity	Compound Name	Medical Parts	Model	Vivo/Vitro	Administration Route^a^	Dose	Duration	Cytokine/Enzyme /Protein	Results	Involved Mechanism	References
Anti-lung cancer	ESPS	Root	Female C57/BLmice	*In vivo*	i.p.	0.12 ml/piece	─	μPA ↓	Intervene of lung cancer	PAI-1 ↓	Zhang (2001)
	ESPS	Root	H446 cells	*In vitro*	—	240,480,960 μg/ml	48 h	—	Cell proliferation ↓	P53, Bax**↑**, Bcl-2 expression ↓ Apoptosis	Zhao (2018)
	ESPS	Root	H446 cells	*In vitro*	—	240,480,960 μg/ml	48 h	TNF-α, IL-1	Cell proliferation ↓	P53,Bax**↑**,Bcl-2**↓**p-38 expression EPK pathway, Apoptosis	Zhao (2018)
	ESPS	Root	H446 cells	*In vitro*	—	240,480,960 mg/l	48 h	—	G2/M arrest ↓	EPK MAP kinase pathways	Zhao (2010)
	ESPS	Root	NSCLC NCI-H520	*In vitro*	—	10,20,40,80,160, 320 mg/ml	12,24,48 h	MMP2, MMP9, FN1, wnt3a	Proliferation, metastasis ↓	Wnt/β-catenin pathways	Sun et al. (2019)
	ASC-1	Root	A-549 cells	*In vitro*	—	ASC-1 35.7 μM	11 days	—	Cell viability ↓	—	Wang, (2006)
	ASA-1	Root	—	—	—	ASA-1 125 μM	—	—	—	—	—
	ASH-1	Root	—	—	—	ASH-1 163.5 μM	—	—	—	—	—
Anti-liver cancer	ESPS	Root	Mice, HEPA cells	*In vivo*	s.c.	0.34 g/kg /d	10 days	—	The life of mice **↑**	—	Chen (1984)
	ASC-1	Root	Male Kunming mice,	*In vivo*	i.g.	2 mg/piece	11 days	ASC-1 prevent seed tumors	—	—	Wang, (2006)
	ASA-1	Root	—	H22 cells	i.p.	0.04 mg/piece	—	—	ASA-1no anti-tumor effect	—	—
	ESPS	Root	Kunming mice,	*In vivo*	i.g.	50,100,200 mg/kg/d	10 days	TNF-α,TNF-γ,	Survival days **↑**,regulate cytokines	—	Meng, (2018)
	—	—	H22cells	—	—	—	—	IL-2, IL-12	—	—	—
	PAS	Root	C57BL/6 mice	*In vivo*	i.p.	10 mg/kg /d	14 days	IL-2R, LAK	LAK activity **↑**, splenocyte **↑**	—	Cao and Du, (1993)
	ESPS	Root	Male Wister rat	*In vivo*	i.g.	20%	70 days	γ-GT ↑	Inhibit tumor	—	Zhang et al. (1997)
	ESPS	Root	HepG2 cells	*In vitro*	—	0,10,20,40,80 mg/L	48 h	—	Apoptosis**↑**, G0/G1 phase arrest in	Wnt/β-catenin pathway	Wang et al. (2016)
Anti-other tumors	ESPS	Root	Kunming mice,	*In vivo*	i.g.	50,100,200 mg/kg/d	10 days	IL-2,IL-12,INF-γ	Cell growth ↓	Apoptosis	Meng et al. (2018)
	ESPS	Root	Kunming mice,	*In vivo*	i.g.	50,100,200 mg/kg/d	10 days	TNF-α,TNF-γ,	Survival days **↑**, regulate cytokines	Immunomodulation	Meng, (2018)
	—	—	U14 cells	—	—	—	—	IL-2, IL-12	—	—	—
	ESPS	Root	Hela cells	*In vitro*	─	1,2,4,8,16 mg/ml	24,48,72 h	─	Cell proliferation ↓	Bax expression **↑** , Apoptosis	Jin (2014)
	ESPS	Root	Hela cells	*In vitro*	─	1,2,4,8,16 mg/ml	24,48,72 h	─	Cell proliferation ↓	Survivin expression ↓, Apoptosis	Jin (2016)
	ESPS	Root	Kunming mice,	*In vivo*	i.g.	50,100,200 mg/kg /d	10 days	TNF-α,TNF-γ,	Survival days **↑**, regulate cytokines	Immunomodulation	Meng, (2018)
	—	—	S180cells	—	—	—	—	IL-2, IL-12	—	—	—
	ESPS	Root	Mice S37,S180 cells	*In vivo*	s.c	0.34 g/kg/d	10 days	—	Excited reticuloendothelial system	Immunomodulation	Chen (1984)
	ESPS	Root	S180cells	*In vitro*	—	1,10,100,500,	24 h	—	Cell proliferation ↓	Apoptosis	Tong (1994)
	—	—	—	—	—	1000 mg/l	—	—	—	—	—
	ESPS	Root	Hep 2 CD133	*In vitro*	—	100 mg/l	24,48,72 h	Bcl-2,PD-L1	CD133 stem cells ↓	Apoptosis	Chen et al. (2019)
Immuno-modulatory	PES-W	Root	Female C57BL mice	*In vivo*	i.p.	100 mg/kg /d	5 days	—	Immunity**↑**, hyperplasia of	Immunomodulation	Xu (1983)
	PES-A	—	—	—	—	—	—	spleen and thymus ↑	—	—	—
	PES-B	—	—	—	—	—	—	—	—	—	—
	PES-W	Root	Female C58BL mice	*In vivo*	i.p.	100 mg/kg/d	4 days	─	The phagocytic function of mice	Immunomodulation	Xu (1983)
	PES-A	—	—	—	—	—	—	—	Peritoneal macrophages ↑	—	—
	PES-B	—	—	—	—	—	—	—	—	—	—
	ESPS	Root	Mice	*In vivo*	i.p.	30 mg/kg/d	3 days	—	Sleep time**↑**, number of thymus ↓	Immunomodulation	Chen (1984)
	ESPS	Root	Mice	*In vivo*	i.p.	30 mg/kg /d	3 days	—	Sleep time↑, white blood cells**↑**	Immunomodulation	Chen (1984)
	ESPS	Root	Mice	*In vivo*	s.c.	0.067 g/kg/d	3 days	—	Formation of antibodies in the spleen **↑**	Immunomodulation	Chen (1984)
	ASII	Root	Mice	*In vivo*	—	—	—	—	Strong phagocytosis **↑**	Immunoregulation	Wang, (1986)
	ASIII	—	—	—	—	—	—	—	—	—	—
	ESPS	Root	Male BALB/c, C57BL/6 mice	*In vivo*	i.p.	100 mg/kg/d	10 days	ConA, LPS	Stimulate T-cell proliferation	—	Xie et al. (1989a)
Immuno-Modulatory	ESPS	Root	Male BALB/c, C57BL/7 mice	*In vivo*	i.p.	100 mg/kg/d	15 days	ConA, LPS	Number of antibody secreting cells**↑** C57BL/7 mice delayed-type hypersensitivity**↑**	Immunomodulation	Xie (1989b)
	ESPS	Root	LACA mice	*In vivo*	i.p.	12.5,25,50,100 mg/kg	4/9 days	—	Humoral immune response**↑**	—	Xu, (1990)
	PES	Root	Female C57BL/JCR	*In vitro*	s.c.	100 mg/kg	4 days	LPS	PES possesses mitogenic activities	Immunomodulation	Shen et al. (1991)
	—	—	mice	—	—	—	—	—	Enhanced LPS, lymphocyte	—	—
	—	—	—	—	—	—	—	—	Transformations ↓, stimulates B-cells	—	—
	ESPS	Root	Kunming mice	*In vivo*	i.g.	25,50,100 mg/kg	7 days	CD3+,CD4+,	The proliferation of splenic	Immunomodulation	Luo (2013a)
	—	—	—	—	—	—	—	CD8+	lymphocytes **↑**,	—	—
	—	—	—	—	—	—	—	—	CD3+CD4+/CD3+CD8+**↑**	Immunomodulation	—
	ESPS	Root	Kunming mice	*In vivo*	i.g.	25,50,100 mg/kg	7 days	CD4+/CD8+,	The body's cellular immune	Immunomodulation	Luo (2013b)
	—	—	—	—	—	—	—	IL-2	function ↑	—	
	ESPS	Root	BALB/c mice	*In vivo*	i.g.	36,25,72.5,145 mg/kg	14 days	─	The phagocytic function of	Immunomodulation	Zhai (2020)
	—	—	—	—	—	—	—	—	immunosuppressed mice **↑**	—	—
	ESPS	Root	BALB/c mice	*In vivo*	i.g.	36,25,72.5,145 mg/kg	7 days	—	The production of hemolysin HC50**↑**	Immunomodulation	Zhai (2020)
	ESPS	Root	BALB/c mice	*In vivo*	i.g.	36,25,72.5,145 mg/kg	7 days	—	The weight of the thymus ↓	Immunomodulation	Zhang, (2015)
	ESPS	Root	BALB/c, DTH mice	*In vivo*	i.g.	36,25,72.5,145 mg/kg	7 days	CD4+/CD8+	Immunosuppressive effect	—	Zhang, (2015)
	ESPS	Root	BALB/c mice	*In vivo*	i.g.	36,25,72.5,145 mg/kg	7 days	IL-2,IL-4,IFN-γ	Cytokine content ↓	Cytokine expression	Zhang, (2015)
	ESPS	Root	BALB/c mice	*In vivo*	i.g.	36,25,72.5,145 mg/kg	14 days	HC50	Sheep red blood cell production	Immune stress	Zhang, (2015)
	—	—	—	—	—	—	—	—	Hemolysin **↑**	—	—
	ESPS	Root	Weaned piglets	*In vitro*	─	0,40,80,160, 320 μg/ml	21 days	NO, iNOS, NF-κB,	Cooperate with ConA to promote	Apoptosis	Han, (2013)
	—	—	—	—	—	—	—	Th1	T- cell proliferation	—	—
	ESPS	Root	Weaned piglets	*In vitro*	─	800 mg/kg	14d	IL-2,IL-6, TNF-α,	Modulate the release of	Cytokine expression	Han et al. (2014)
	—	—	—	—	—	—	—	α-AGP	Pro-inflammatory cytokines	—	—
	ESPS	Root	ICR mice	*In vivo*	i.g.	6,12,24 mg/10g/d	21 days	TNF-α, INF-γ	Regulate the serum hemolysin level,	Apoptosis	Sun et al. (2018)
	—	—	—	—	—	—	—	—	Cell apoptosis ↓ to enhance the	—	—
	—	—	—	—	—	—	—	—	Body's humoral immune function.	—	—
	PES	Root	Mice	*In vivo*	i.p.	125 mg/kg/d	5 days	BSAIgG**↑**	Enhance the defense.	Antioxidant index	Zhu et al. (1982)
Anti-inflammatory	ESPS	Root	Kunming mice	*In vivo*	i.g.	20 mg/kg/d	10 days	IL-1, IL-2,	Improve immune cytokines	Cytokine expression	Meng, (2018)
	—	—	—	—	—	—	—	IL-10, TNF-α	—	—	—
	ESPS	Root	BALB/c mice	*In vivo*	i.g.	36.25,72.5,145 mg/kg	7 days	IL-1, IL-1β, IL-2, IL-4	Number of enzymes ↓,protect liver	Antioxidant index	Zhang (2018)
	CASPs	Root	Wistar male rats	*In vivo*	i.g.	50,100,200 mg/kg	15d	IL-10**↑**, IL-1β, TNF-α**↓**	Inflammatory cytokines**↓**	—	Xie et al. (2015)
	ESPS	Root	Male BALB/c mice	*In vivo*	i.g.	14.5 mg/ml/d	7 days	IL-4, Cyclic GMP	Improve the body’s immunity,	Glutathione metabolism, purine	Yang, (2016)
	—	—	—	—	—	—	—	—	Anti-inflammatory, Antioxidant,	Generation metabolism, cysteine ,	—
	—	—	—	—	—	—	—	—	and supply the body's energy	methionine metabolism pathways	—
	ASPS-1-1	Root	Male BALB/c mice	*In vivo*	i.g.	6.9 mg/ml	7 days	IL-1β, TNF-α, IL-2,	Liver cell damage ↓	—	Yang, (2016)
	ASPS-2-1	—	—	—	—	4.6 mg/ml	—	IL-4, IL-6	—	—	—
	ASPS-3-1	—	—	—	—	2.0 mg/ml	—	—	—	—	—
	ESPS	Root	BALB/c mice	*In vivo*	i.g.	36.25,72.5,145 mg/kg	7 days	IL-2, IL-4,	Secretion and expression of	Cytokine expression	Zhang (2018)
	—	—	—	—	—	—	—	—	inflammatory cytokines ↓	—	—
	ESPS	Root	BALB/c mice	*In vivo*	i.g.	36.25,72.5,145 mg/kg	7 days	MDA, IL-1β, TNF-α,	The activity of inflammatory	Cytokine expression	Zhang et al. (2019)
	—	—	—	—	—	—	—	NO, GSH-Px, SOD,	Cytokines, adhesion factors**↓**,	—	—
	—	—	—	—	—	—	—	ICAM-1, iNOS,	The secretion and expression of	—	—
	—	—	—	—	—	—	—	NF-κB	Inflammatory cytokines**↓**	—	—
	ESPS	Root	KM mice	*In vivo*	i.g.	14.5 mg/ml/d	7 days	IL-2, IL-6	By enhancing the body's immune function, scavenging free radicals, liver cell apoptosis↓, and other mechanisms	Bile secretion, Cysteine metabolism, Ubiquinone and other terpenoid quinone biosynthesis, Citrate cycle regulating the body's cytokines Purine metabolism, Riboflavin metabolism, Primary bile acid biosynthesis, Biosynthesis of unsaturated fatty acids, Cysteine and methionine metabolism	Lu (2016)
Anti-leukemia	ESPS	Root	Mice, L615 cells	*In vivo*	i.p.	0.34 g/kg/d	5 d	—	No significant effect on survival time	Oxidative stress	Chen (1984)
	ESPS	Root	S801, S7811 cells	*In vitro*	—	10 μg/ml	single	—	Improve the ability of cells to produce interferon	Oxidative stress	Yang et al. (1983)
	ESPS	Root	S801,S7811 cells	*In vitro*	—	10 μg/ml	single	—	The life span of the transcribed dry money mRNA**↑** or mRNA inactivation rate**↓**	Apoptosis	Yang et al. (1985)
	ESPS	Root	K562 cells	*In vitro*	—	1,10,100,500, 1000 mg/l	24 h	—	Cell proliferation**↓**	Apoptosis	Tong (1994)
	ESPS	Root	K562 cells	*In vitro*	—	0.405, 0.810, 1.620, 2.430, 3.240 mg/mL	24 h	—	K562 cell apoptosis **↑**	Apoptosis	Luo (2008)
	ASC-1	Root	P-388 cells	*In vitro*	─	35.69 μM	single	—	Mouse white blood cell activity**↓**	—	Wang, (2006)
	ASH-1	—	—	—	—	164.23 μM	single	—	—	—	—
Anti-oxidation	ESPS	Root	·OH, O^2-^	*In vitro*	─	3 mg/l	single	—	Elimate ·OH and O^2-^	Oxidative stress	Meng et al. (2005)
	ESPS	Root	·OH, O^2-^	*In vitro*	─	3 mg/l	single	—	Elimate ·OH and O^2-^	Oxidative stress	Jiao, (2008)
	—	—	—	—	—	—	—	—	Inhibit lipid peroxidation of red blood	—	—
	—	—	—	—	—	—	—	—	cell membrane**↓**	—	—
	ASP-2-1	Leaf	Mice spleen cells	*In vitro*	—	0.2 mg/ml	single	—	Elimate ·OH, O^2-^, DPPH·	Oxidative stress	Chen (2011a)
	ESPS	Root	SD rats hippocampal neurons	*In vitro*	—	1.25, 2.5, 5, 10 g/ml	7 days	SOD,NOS,MDA	Improve cell resistance to oxidative stress damage	Bax expression **↓**,Bcl-2 expression **↑**	Diao (2008a)
	ESPS	Root	SD rats hippocampal neurons	*In vitro*	—	1.25, 2.5, 5, 10 μg/ml	7 days	—	Improve the anti-apoptotic ability	Bax expression **↓**,Bcl-2 expression **↑**	Diao (2008b)
	ESPS	Root	SD rats hippocampal neurons	*In vitro*	—	1.25, 2.5, 5, 10 μg/ml	7 days	LDH,SOD,MDA	Improve the anti-apoptotic ability	P53 expression	Diao (2009a)
	ESPS	Root	SD rats hippocampal	*In vitro*	—	1.25, 2.5, 5, 10 μg/ml	7 days	NO, iNOS mRNA**↓**	The expression of iNOSmRNA**↓**	iNOSmRNA expression	Diao (2008c)
	ESPS	Root	SD rats hippocampal	*In vitro*	—	1.25, 2.5, 5, 10 μg/ml	7 days	—	Anti-apoptotic ability **↑**	P53 expression **↓**	Diao (2009b)
	ESPS	Root	SD rats hippocampal neurons	*In vitro*	—	1.25, 2.5, 5, 10 μg/ml	7 days	SOD, CAT, GSH-Px, MDA	Activity of Antioxidant **↑**	Antioxidant index	Diao (2008d)
	ESPS	Root	SD rats hippocampal neurons	*In vitro*	—	1.25, 2.5, 5, 10 μg/ml	7 days	MDA, ROS, H_2_O_2_	The content of oxygen free radicals	Oxidative stress **↓**	Diao (2009c)
	ESPS	Root	SD rats hippocampal neurons	*In vitro*	—	1.25, 2.5, 5, 10 μg/ml	7 days	SOD, GSH-Px, OGG1mRNA	OGG1mRNA expression **↑**	OGG1mRNA expression	Diao (2009d)
	CASPs	Root	Wistar male rats	*In vitro*	i.g.	50, 100, 200 mg/kg	15 days	SOD, GSH-Px**↑**, MDA**↓**	Oxidative damage **↓**	Oxidative stress	Xie et al. (2015)
	ESPS	Root	SD rats hippocampal neurons	*In vitro*	—	2.5,5,10 μg/ml	7 days	Fas, Fasl**↓**	Cell apoptosis**↓**	Fas,Fasl**↓**, Apoptosis	Diao et al. (2010a)
	ESPS	Root	SD rats hippocampal neurons	*In vitro*	—	2.5,5,10 μg/ml	7 days	—	Gene expression**↓**, the activity**↑**	C-fos,p53 expression	Diao et al. (2010b)
	ESPS	Root	SD rats hippocampal neurons	*In vitro*	—	2.5,5,10 μg/ml	7 days	NF-κB**↓**	Gene expression**↓**	NF-κB pathways	Diao et al. (2010c)
	ESPS	Root	SD rats hippocampal neurons	*In vitro*	—	2.5,5,10 μg/ml Caspase-3 mRNA	24 h	Caspase-3	Resist oxidative stress damage	Oxidative stress	Liu et al. (2013)
Other biological activities	ESPS	Root	Kunming mice	*In vivo*	—	300 mg/kg	14 days	DAO, occludin-1, zonula occludens-1	Improves intestinal integrity	TLR4/NF-κB signaling pathway	Han et al. (2016a)
	ESPS	Root	Kunming mice	*In vivo*	i.g.	300 mg/kg	14 days	Occludin-1, HSP70	Gene mRNA expression of epidermal growth factor and its receptor ↑	mRNA expression	Han et al. (2016b)
	ESPS	Root	Male Wistar rat	*In vivo*	i.g.	50,100,200 mg/kg	10 days	—	Protect the body weight of irradiated rats	—	Mo (2012a)
	ESPS	Root	Male Wistar rat	*In vivo*	i.g.	50,100,200 mg/kg	10 days	WBC, RBC, PLT	Protect peripheral blood cells from radiation exposure	—	Mo (2012b)
	ESPS	Root	Adult flies	*In vivo*	i.g.	15,30 mg/ml	5 days	—	Protects the intestinal tract from DSS	EGFR, JNK, Notch pathways	Zhang et al. (2020a)
	ESPS	Root	BALB/C mice	*In vivo*	i.g.	300 mg/kg	7 days	MLCK, TJ, NF-κB, LPS	Associated with inhibition of the NF-κB/MLCK pathway	NF-κB/MLCK pathway	Han et al. (2017)
	ESPS	Root	Kunming mice	*In vivo*	i.g.	50,100,200 mg/kg	7 days	IL-6, TNF-α, IL-1β	Improve immune organ index and regulate cytokine levels	—	Pan (2019b)
	ESPS	Root	Male SD rat	*In vivo*	i.g.	100 mg/kg	20 days	—	Neuroprotection, anti-apoptosis	Anti-apoptosis	Lu (2015)
	ESPS	Root	Male kunming mice	*In vivo*	i.g.	450,150 mg/kg/d	14 days	—	Ameliorate energy metabolism, creatinine, improve the level of serum the breakdown of protein**↓** free radical content of the body	Energy metabolism	Han, (2007)
	ESPS	Root	Kunming mice	*In vivo*	i.g.	50,100,200 mg/kg/d	5 days	IL-6, TNF-α	Inhibit swelling, improve immune organs, immune cytokines	Immunomodulation	Meng, (2018)
	ESPS	Root	Female SD rat	*In vivo*	s.c.	100 mg/kg/d	2/5 days	sICAM-1, TGF-β1, LYM, RT, GLU, LDH	Improve pleural effusion	Immunomodulation	Meng, (2018)
	ESPS	Root	Kunming mice	*In vivo*	i.g.	50,100,200 mg/kg/d	7 days	IL-1β, TNF-α	Improve immune organs, immune cytokines	Immunomodulation	Meng, (2018)
	ASP	Root	Male Wistar rats	*In vivo*	i.g.	200 mg/kg	28 days	SOD, GPX, TC, TG, TBARS	Adjust the pathophysiological	Antioxidant index parameters of diabetic rats	Fu et al. (2012)
	ESPS	Root	Kunming mice	*In vivo*	i.g.	50,100,200 mg/kg	5 days	SOD, GSH-PX,	Improve the Antioxidant capacity CAT, MDA	Antioxidant index caused by D-Gal	Meng, (2018)
	ESPS	Root	Female SD rat	*In vivo*	i.p.	50,100,200 mg/kg/d	5 days	SOD, MDA, AChE, ChAT	Regulate enzyme activity in brain tissue and serum	Immunomodulation	Meng, (2018)
	ANP	Buds	RAW-Blue TM cells	*In vitro*	—	—	—	—	Promoted by the interaction through	—	Lee et al. (2015)
	AAP	—	(RAW 264.7)	—	—	—	—	—	the membrane receptors	—	—
	PEA	Root	—	*In vitro*	i.g.	1.75 g/kg	24 h	CRP	Suppress hypoglycemia and	Inflammation inflammation to relieve alcohol	Bang et al. (2015)

a i. g. intragastric administration; i. v. intravenous injection; i. p. intraperitoneal injection; s. c. subcutaneous injection.

## Conclusions and Future Perspectives

Consult the related literature and data about *E. senticosus*, we can find that the related studies mainly focused on small molecule components, which ignored macromolecular substances such as protein, polysaccharides, pectin, and cellulose. As the effective ingredient of *E. senticosus*, polysaccharide possess a wide range of pharmacological effects with the advantages of non-toxic, two-way immune regulation, and has great potential for clinical application. However, research on polysaccharides has increased resistance due to its high molecular weight, complex structure, low content, difficulty of separation and low purity. The research work of its future perspectives is mainly reflected in the following aspects.

1) From aspects of efficient extraction, the existing methods expose the disadvantages of low yield and long extraction time, and therefore cannot be industrialized in mass production. Instead, a wide range of green environmental technologies such assisted extraction with enzymes and chelating agents have not been studied and applied in the extraction of ESPS.
2) As for the resource utilization, the residues of herbs and fruits after extraction of small molecules are rich in a large number of macromolecular substances, such as pectin, polysaccharides and cellulose. Thus, recirculated extraction is conducive to the sustainable development and maximum utilization of traditional Chinese medicine. It has been reported that relative high yield of sugars and pectin was separately extracted from residues of *Ginseng* (Zhang et al., 2020b) and Sweet Potato Peels (Hamidon et al., 2020). Meanwhile, Chen et al. found that a large amount of ESPS could be extracted from petioles, leaves and fruits (Chen, 2009). However, no literature on the reuse and expansion of *E. senticosus* resource were found. Collectively, extraction of ESPS from the residue and other medical parts will provide us future research direction.
3) Recently, the rapid development and application of biological macromolecules in new drug delivery system and edible film provide people new directions and technologies for pharmaceutical and food preservation. If ESPS could pack or interact with other ingredients (flavonoids, saponins, protein) and then act as drug delivery system is relatively empty areas.
4) At present, several products derived from *E. senticosus* such as capsules, wine and tea have long been developed and marketed in view of their excellent health-care function and clinical therapeutic effect. However, ESPS has not been put into clinical use in view of the absence of quality control standards and reference substance. How to establish a comprehensive quality management system is a problem worth thinking about.

In conclusion, health has received more and more attention with the development of science and the improvement of living standard, and the exploitation of functional food has become a research hotspot and development trend. *E. senticosus,* a homology of medicine and food as the main raw materials, has displayed the most economic and developing value. Meanwhile, the emergence of ESPS can better inherit, innovate and develop traditional Chinese medicine culture and products. It is believed that ESPS will be widely applied soon in the fields of clinical and functional food with the deepening of research and the maturity of technology.

## Glossary

AchE: acetylcholinesterase
AG: arabinogalactans
ALP: Alkaline phosphatase
ALT: alanine aminotransferase
AST: aspartate aminotransferase
Ara: arabinose
ChAT: choline acetyl-transferase
ConA: Concanavalin A
CVD: cardiovascular disease
CTX: cyclophosphamide
EGFR: epidermal growth factor receptor
ERK: regulated protein kinase
ESPS: Eleutherococcus senticosus polysaccharides
Fuc: fucose
Fru: fructose
FT-IR: Fourier transform infrared spectroscopy
Gal: galactose
GC: gas chromatography
Glc: glucose
GalA: galacturonic acid
GlcA: glucuronic acid
GSH: glutathione
GSH-Px: glutathione peroxidase
HPLC: high-performance liquid chromatography
i.g.: intragastric administration
i.v.: intravenous injection
i.p.: intraperitoneal injection
IFN: interferon
ICAM: intercellular adhesion molecule
iNOS: inducible nitric oxide synthase
IL: interleukin
JNK: c-jun N-terminal kinase
LAK: lymphokine-activated killer cell
LC: liquid chromatography
LC: Lung cancer
LPS: lipopolysaccharide
Man: mannose
MAPK: mitogen-activated protein kinase
MDA: malonaldehyde
MMP: mitochondrial membrane potential
MS: mass spectrometry
NF-κB: nuclear factor-kappa B
NSCLC: non-small cell lung cancer
NMR: nuclear magnetic resonance
NO: nitric oxide
OGG1: 8-hydroxyguanine DNA glycosidase
PLT: platelets
Rha: rhamnose
RBC: red blood cells
RG: rhamnogalacturonan
Rib: ribose
SCLC: small cell lung cancer
s.c.: subcutaneous injection
SFE: supercritical fluid extraction
SOD: superoxide dismutase
TBARS: thiobarbituric acid reactive substances
TCA: trichloroacetic acid
TLR: toll-like receptor
TC: serum total cholesterol
TG: triglyceride
TNF: tumor necrosis factor
UAE: ultrasound-assisted extraction
WBS: white blood cells
XGA: xylogalacturonan
Xyl: xylose.
